# Supplementary material for: Educational attainment and differences in relative survival after acute myocardial infarction in Norway: a registry-based population study
Source: BMJ Open. 2017 Aug 28;7(8):e014787. doi: 10.1136/bmjopen-2016-014787 (PMC5724085; doi:10.1136/bmjopen-2016-014787)
Supplement: Supplementary file 1 [file bmjopen-2016-014787supp001.pdf]

ONLINE SUPPLEMENTARY MATERIAL

S1. Alternative calculation of education-specific expected survival

In our main analysis, we used Poisson regression to predict expected survival for the years 2011–2013. For comparison, we also applied an alternative method, based on an approach of Dickman et al. [1]. In this alternative method, all-cause mortality data obtained from the Human Mortality Database were adjusted based on our data containing educational levels.[2] Let $\mu_{g,t}$ denote the rate of all-cause mortality in year *t* and educational group *g*, and for brevity omit subscripts for age and sex. We then employed the following expression,

$$\mu_{g,t} = \mu_{c,t} \left(\frac{\mu_{g,t^*}}{\mu_{c,t^*}}\right)$$

where values for the whole country have group-subscripts replaced by *c*, and where t^* indicates that estimates for the years 2008–2010 were used. The values $\mu_{c,t}$ were obtained from the Human Mortality Database for each year *t = 2008, …, 2013*, while the standardized mortality ratio $(\mu_{g,t^*} / \mu_{c,t^*})$ was taken from the obtained life tables on the general population from Statistics Norway for the combined period 2008–2010. The resulting estimates were nearly identical to those in our main analysis.

S2. Correction for infarction-related mortality and sensitivity analysis

Expected survival was corrected for mortality related to acute myocardial infarction, using the formula

$$p^* = p^{(1-\alpha)}$$

where p^* is the corrected survival probability, p is the observed survival probability, and α is the proportion of deaths due to acute myocardial infarction.[3] Because the necessary data were unavailable, we could not correct for α_g directly. Nonetheless, by emulating the relationship for overall cardiovascular mortality (ICD-10 I00–I99), as represented by λ, we obtained an approximate estimator:

$$\frac{\alpha_{g,t}}{\alpha_{c,t}} \approx \left(\frac{\lambda_{g,t}}{\mu_{g,t}}\right) \Big/ \left(\frac{\lambda_{c,t}}{\mu_{c,t}}\right) \quad \Leftrightarrow \quad \alpha_{g,t} \approx \alpha_{c,t} \left(\frac{\lambda_{g,t^*}}{\lambda_{c,t^*}}\right) \Big/ \left(\frac{\mu_{g,t^*}}{\mu_{c,t^*}}\right)$$

In the rightmost expression we used nationwide mortality data for $\alpha_{c,t}$.[4] The subscript t^* indicates that estimates for 2000–2010 were used for λ and μ.[5] The complex fraction was assumed to equal 1 in our main specification, and varying it based on previous findings had only a minor influence on the results.[5]

REFERENCES FOR THE SUPPLEMENTARY MATERIAL

1. Dickman PW, Auvinen A, Voutilainen ET, Hakulinen T. Measuring social class differences in cancer patient survival: is it necessary to control for social class differences in general population mortality? A Finnish population-based study. *J Epidemiol Community Health*. 1998;52:727–34.
2. Human Mortality Database. University of California, Berkeley (USA), and Max Planck Institute for Demographic Research (Germany). Available at www.mortality.org (data downloaded on November 10, 2015).
3. Talback M, Dickman PW. Estimating expected survival probabilities for relative survival analysis--exploring the impact of including cancer patient mortality in the calculations. *Eur J Cancer* 2011;47:2626–32.
4. Norwegian Institute of Public Health. Table: [D1: Deaths by sex, age, and detailed cause of death] D1: Dødsfall etter kjønn, alder og detaljert dødsårsak. Available at statistikkbank.fhi.no/dar/ (data downloaded on December 1, 2015). Norwegian.
5. Strand BH, Steíngrimsdóttir ÓA, Grøholt EK, Ariansen I, Graff-Iversen S, Næss Ø. Trends in educational inequalities in cause specific mortality in Norway from 1960 to 2010: a turning point for educational inequalities in cause specific mortality of Norwegian men after the millennium? *BMC Public Health* 2014;14:1208.
