# Supplementary material for: Educational attainment and differences in relative survival after acute myocardial infarction in Norway: a registry-based population study
Source: BMJ Open. 2017 Aug 28;7(8):e014787. doi: 10.1136/bmjopen-2016-014787 (PMC5724085; doi:10.1136/bmjopen-2016-014787)
Supplement: Author's manuscript [file bmjopen-2016-014787.draft_revisions.pdf]

BMJ Open is committed to open peer review. As part of this commitment we make the peer review history of every article we publish publicly available.

When an article is published we post the peer reviewers' comments and the authors' responses online. We also post the versions of the paper that were used during peer review. These are the versions that the peer review comments apply to.

The versions of the paper that follow are the versions that were submitted during the peer review process. They are not the versions of record or the final published versions. They should not be cited or distributed as the published version of this manuscript.

BMJ Open is an open access journal and the full, final, typeset and author-corrected version of record of the manuscript is available on our site with no access controls, subscription charges or pay-per-view fees (http://bmjopen.bmj.com).

If you have any questions on BMJ Open's open peer review process please email info.bmjopen@bmj.com

BMJ Open

Educational attainment and differences in relative survival after acute myocardial infarction in Norway

Journal:	*BMJ Open*
Manuscript ID	bmjopen-2016-014787
Article Type:	Research
Date Submitted by the Author:	19-Oct-2016

Complete List of Authors:	Klitkou, Søren; University of Oslo, Health Management and Health Economics<br>Wangen, Knut ; University of Oslo, Health Management and Health Economics

**Primary Subject Heading**:	Public health
Secondary Subject Heading:	Cardiovascular medicine
Keywords:	Coronary heart disease < CARDIOLOGY, Record linkage, Health inequalities, Education, Mortality

SCHOLARONE™
Manuscripts

For peer review only

Version: October 18th, 2016

Title:

Educational attainment and differences in relative survival after acute myocardial infarction in Norway

Søren T. Klitkou[1], Knut R. Wangen[1]

[1] Department of Health Management and Health Economics, Institute of Health and Society, University of Oslo, Oslo, Norway

Corresponding author:

Søren Toksvig Klitkou

Department of Health Management and Health Economics

University of Oslo

Postal address: Postbox 1089 Blindern, NO - 0317 Oslo, Norway

Phone number: +47 228 45 028

Fax number: +47 228 45 091

Email address: s.t.klitkou@medisin.uio.no

Key words: Coronary heart disease, Record linkage, Health inequalities, Education, Mortality

Word count excluding Tables, Abstract, Strengths and limitations of this study, What is known on this topic, What this study adds, Acknowledgments, References, and Supplementary files: 3027

Number of tables = 3; Figures = 1; References = 40

Article type: observational study (research report)

For peer review only - http://bmjopen.bmj.com/site/about/guidelines.xhtml

Abstract

Background: Although there is a broad societal interest in socioeconomic differences in survival after an acute myocardial infarction, only a few studies have investigated how such differences relate to the survival in general population groups. We aimed to investigate education-specific survival rates after acute myocardial infarction and to compare these rates with the survival of corresponding groups in the general population.

Methods: Our study included the entire population of Norwegian patients admitted to hospitals for acute myocardial infarction during 2008–2010, with a six-year follow-up period. Patient survival was measured relative to the expected survival in the general population for three educational groups: primary, secondary, and tertiary. Education-, sex-, age-, and calendar-year-specific expected survival rates were obtained from population life tables and adjusted for the presence of infarction-related mortality.

Results: Six-year patient survivals were, respectively, 56.3% (55.3-57.2) and 65.5% (65.6-69.3) for the primary and tertiary educational groups (95% confidence intervals). Also six-year relative survival was markedly lower for the primary educational group: 70.2% (68.6 - 71.8) versus 81.2% (77.4 - 84.4). Throughout the follow-up period, patient survival tended to remain lower than for members of the general population with the same educational background.

Conclusion: Both patient survival and relative survival after acute myocardial infarction are positively associated with educational level. Our findings may suggest that secondary prevention has been more effective for the highly educated.

For peer review only

For peer review only - http://bmjopen.bmj.com/site/about/guidelines.xhtml

Strengths and limitations of this study

- The relative survival framework made possible the comparison of patient survival to population survival for a national cohort of AMI patients and to distinguish how groups defined by educational level fared compared to their group in the population.
- The use of nationwide registry data from patients and the general population give findings that are representative for Norway for the years of diagnosis from 2008 to 2010 with a maximum follow-up of six years until the end of 2013.
- The study has low levels of missing data on education (<2%) and identical definitions of educational attainment in patient and population groups.
- It is not possible from the study to attribute causality to the age- and sex-standardized differences between educational groups regarding patient survival, the expected survival in the general population, and the relative survival of AMI patients.

For peer review only

For peer review only - http://bmjopen.bmj.com/site/about/guidelines.xhtml

What is known on this topic

Survival after acute myocardial infarction continues to differ considerably across educational groups. However, it is less clear how the time course of survival rates among patients from different educational groups relate to the time course of survival in the general population.

What this study adds

After acute myocardial infarction, the survival of patients with the highest educational level remained comparatively closer to the survival of members of the general population with the same educational background. This suggests that the highest education group retains more of the conditional life expectancy of the general population than groups with lower education levels.

In the period extending beyond the first year of follow-up, patient survival was lower than survival in the general population. This may inform decision analytic models on the health benefits and cost consequences of interventions for cardiovascular disease.

The higher relative survival ratios that were observed among highly educated individuals may have implications for the health services that are offered after acute myocardial infarction. For example, individuals with lower education levels could benefit from closer medical follow-up and greater motivation to undertake lifestyle changes.

For peer review only

For peer review only - http://bmjopen.bmj.com/site/about/guidelines.xhtml

INTRODUCTION

Socioeconomic differences in survival after acute myocardial infarction (AMI) are of broad societal interest, especially because ischemic heart disease is the single most important cause of death, life-years lost prematurely, and life-years lost to disability [1 2]. The causes of heart disease and AMI continue to be associated with lifestyles [3 4] and life conditions [5-9], resulting in substantial socioeconomic differences in the rates of disease onset and mortality, which correlate with measures such as education level, income, and occupational class [10-12]. Inequalities in mortality have widened in both absolute and relative terms, a public health phenomenon that has been described as a great disappointment in highly developed welfare states [13]. This widening inequality is in large part attributable to the persistent relationship between socioeconomic status and mortality from cardiovascular disease.

Patient survival following AMI is commonly assessed using overall patient survival [14]. This can be appropriate from a clinical perspecitve, say when comparing treatment strategies. However, AMI occurs more frequently in age groups with elevated background mortality and it is therefore of interest to measure patient survial relative to the survival of the general population. In relative survival this comparison is accomplished by life-tables for population survival [15].

Dickman et al. emphasized the importance of stratifying the general population life-tables according to the adopted measure of socioeconomic status when the aim is to compare separate social groups [16]. The relative survival approach was later refined to situations where the disease of interest had a substantial influence on population survival – typically when a relatively common disease causes high mortality [17]. The principle of the correction was to adjust the population survival so resembling survival in a population *without* the disease of interest.

Socioeconomic and demographic gradients in relative survival have been studied previously for AMI patients. A Singaporean study found lower relative survival among the Malayan minority, as compared with the Chinese and Indian ethnic groups [18], while a Spanish study reported no association between relative survival and a social deprivation index [19]. However, neither these nor similar studies of cardiovascular disease [18-22] applied the mentioned refined approach.

For peer review only - http://bmjopen.bmj.com/site/about/guidelines.xhtml

In the context of this prior research, our aim was to investigate relative survival ratios after AMI for patients with different educational backgrounds in Norway. We used registry data that covered the entire Norwegian population. A single-payer healthcare system and a tuition-free higher education system are provided by the public sector in Norway. In principle, this offers the whole population equal access to healthcare and education. Educational inequalities in 28-day and 1-year AMI survival have been reported previously [23], but no comparison with the general population has been published to date. Additionally, in contrast to previous studies on relative survival for cardiovascular disease [18-22], we have accounted for AMI mortality in the general population.

METHODS

Data

For the current study, we considered all hospital admissions for AMI (ICD-10 I21-I22) that were included in the Norwegian National Patient Registry for the period 2008–2010. Information on date of death was obtained from the Causes of Death Registry for the period 2008–2013. This registry covers all deaths in Norway and deaths of Norwegians who die abroad. Data on educational level were obtained from the National Database on Education.

We identified 35,045 patients aged 40–94 years with an AMI diagnosis in the patient registry. Patients who were younger or older at the time of infarction were excluded because of small numbers of patients in these groups. For patients with multiple AMI episodes, the first episode was included in the analysis. Educational level was defined as the highest completed level of education, and was categorized as follows: primary (6–9 years of schooling), secondary (10–12 years of schooling) or tertiary (university or college degree). We excluded 637 patients with missing information on attained education, leaving a total sample of 34,408 patients. In all 12,135 persons died during the course of follow-up. The observed number of person-years was 116,269, with an average follow-up of 3.4 years per person.

For peer review only - http://bmjopen.bmj.com/site/about/guidelines.xhtml

To calculate relative survival ratios, we obtained life tables from Statistics Norway for the calendar years 1999–2010, which included data by educational level, sex, and 1-year age interval. The life tables included all deaths and person-years, permitting the calculation of expected survival. Importantly for our application, the patients and the general population were stratified using identical definitions of educational groups [16 24].

Statistical analyses

For a given calendar year and population stratum (educational level, sex, and age), relative survival was defined as the ratio of survival among the AMI patients to the expected survival for the general population.

Expected survival for the years 2008–2010 was obtained directly from the life tables covering the years 1999–2010. However, we used model-based predicted survival for the years 2011–2013 because data were lacking for these years. The predicted survival estimates were obtained using Poisson regression analyses of mortality rates, as conducted separately for each combination of educational level and sex. Each Poisson regression included a linear trend for calendar year and restricted cubic splines to represent a possible non-linear age effect, where the number of knots used was decided by Akaike Information Criterion. We conducted two sensitivity analyses for the estimates of expected survival: first, by following the approach suggested by Dickman et al. [16], and second, by forwarding the estimates from the last available year of data (2010). For the approach of Dickman et al. [16], we obtained life tables for Norway for 2008–2013 from the Human Mortality Database [25], which were stratified by year, age, and sex. To obtain education-specific survival estimates, we used the above mentioned 1999–2010 population life table to estimate standardized mortality ratios by educational level for the years 2008–2010, and then applied these ratios to the Human Mortality Database life tables for 2008–2013 (see supplementary description S1 for further details).

Life tables for the general population include significant proportions of deaths due to AMI, particularly at older ages [26]. We therefore adjusted the expected survival for the proportion of deaths due to AMI using Talbäck and Dickman's correction formula [17]. To this end, we obtained data from the Norwegian Institute of Public Health [26] on the sex and age-group specific proportions of deaths

For peer review only - http://bmjopen.bmj.com/site/about/guidelines.xhtml

due to AMI. As these data did not include educational level, we performed a sensitivity analysis based on published comparisons of cardiovascular mortality (ICD-10 I00–I99) with overall mortality [27] (see supplementary description S2 for further details).

We summarized survival differences by level of education at the end of follow-up, as stratified by 5-year age groups. To investigate the time course of survival since AMI, we calculated survival rates for the first month and each year thereafter. For each of these time points, we calculated relative survival (using the Ederer II method [28 29]) by directly standardizing the three educational groups to the overall age and sex distribution. The standardized survival rate was defined as the weighted average of the survival rates in each age and sex group, where the weights equalled the proportions of patients in each group. Finally, we performed a continuous-time analysis of survival rates, in which the time course of survival since AMI was estimated using the Kaplan-Meier and the time course of expected survival was estimated using the Ederer I method [28], as standardized by reweighting to the overall age and sex distribution. Stata 14 (StataCorp, College Station, TX, USA) and the Strs-procedure [28] were used for the calculations in the tables. Additionally, we used R version 3.1.2 and the Survival package for continuous-time estimates.

RESULTS

The average age at hospitalization was 71 years, and 36% of the patients were women. Of the 34,408 patients, 14,360 had completed primary education, 15,527 had completed secondary education, and 4,521 had completed tertiary education.

Table 1 shows the overall patient survival rates at six years of follow-up, as well as the survival rates that would have been expected if the patients had experienced the mortality rates of the general population. Finally, the table also presents the ratio of these two survival rates, which is known as the relative survival ratio. These quantities are calculable for the group of patients diagnosed in 2008 for whom follow-up is six years. There are no indications of a socioeconomic gradient to the differences in relative survival for the age groups 40–49 and 50–54 years and, if anything, the pattern is mixed in these groups. However, the expected survival rate is always higher for higher levels of education.

For peer review only - http://bmjopen.bmj.com/site/about/guidelines.xhtml

From age 55 onwards, patient survival clearly differs according to educational level. For example, among patients aged 55–59 years, the six-year survival rate was 82.7% of those with a primary education level, 87.5% for those with a secondary education level, and 93.3% for those with a tertiary education level. It is notable that these five- and ten-percentage point differences were observed at rather young ages. Whereas the group of patients with a primary education level had an expected survival rate of 95.2%, which translates to a relative survival ratio of 86.9%, the corresponding relative survival ratios for secondary and tertiary education levels were 90.0% and 95.0%, indicating that these patients had survival rates that remained closer to the survival rates of members of the general population with similar educational backgrounds. The relative survival in the primary education group at younger ages appeared to be similar to the relative survival in the tertiary education group at older ages: Relative survival was 86.9% for both patients aged 55–59 with a primary education level and patients aged 65–69 with a tertiary education level. Similarly, relative survival was 36.7% for patients aged 80–84 with a primary education level, and 36.2% for patients aged 90–94 with a tertiary education level.

For peer review only

For peer review only - http://bmjopen.bmj.com/site/about/guidelines.xhtml

Table 1. The 6-year survival rates of patients after acute myocardial infarction, according to age and education level. Observed patient survival rates, expected survival rates,[1] and relative survival ratios are shown.

	Primary education				Secondary education				Tertiary education			
**Age, years**	**N**	**PS**	**ES**	**RS**	**N**	**PS**	**ES**	**RS**	**N**	**PS**	**ES**	**RS**
**40-49**	872	88.7	98.4	90.2	966	92.8	99.2	93.6	396	89.5	99.5	89.9
**50-54**	693	88.6	97.0	91.4	1161	88.5	98.2	90.1	400	90.9	99.0	91.9
**55-59**	792	82.7	95.2	86.9	1621	87.5	97.2	90.0	550	93.3	98.2	95.0
**60-64**	1179	73.1	93.5	78.2	2199	79.9	95.3	83.8	704	86.2	97.2	88.8
**65-69**	1221	63.9	90.3	70.7	1712	72.2	92.9	77.6	597	82.6	95.0	86.9
**70-74**	1514	54.7	86.4	63.4	1649	60.3	89.3	67.5	474	67.8	92.2	73.5
**75-79**	1962	40.8	78.7	51.8	1685	45.1	81.4	55.4	438	57.3	84.9	67.4
**80-84**	2500	24.2	66.0	36.7	1931	28.9	70.1	41.2	424	31.5	73.9	42.6
**85-89**	2394	13.0	50.2	25.8	1727	15.5	53.0	29.2	378	23.7	57.0	41.6
**90-94**	1233	7.4	31.4	23.6	876	8.2	33.1	24.8	160	13.9	38.3	36.2

[1] Expected survival has been corrected for mortality from acute myocardial infarction. PS: patient survival, ES: expected survival, RS: relative survival. The patient survival estimates are by year six and were based on the subsample of patients diagnosed with AMI in 2008 with complete follow-up.

For peer review only

For peer review only - http://bmjopen.bmj.com/site/about/guidelines.xhtml

Table 2 presents the time course of patient survival. The six-year survival was 56.3% and 67.5 % for patients with primary and tertiary education, respectively. Accounting for the expected survival in the general population, Table 3 presents the time course of relative survival ratios over the follow-up period. In each year, the AMI patients fared worse than their counterparts in the general population. Survival decreased substantially during the first year since AMI, for which the relative survival ratios were 83.0%, 84.6%, and 87.0% in the primary, secondary, and tertiary education groups, respectively. By year six, ratios of relative survival were 70.2%, 73.1%, and 81.2% in these groups—the difference in relative survival had become markedly larger. For the primary and secondary education groups, the decline in relative survival was monotonic over the six-year period. In contrast, for the tertiary education group, the relative survival ratio appeared to stabilize at approximately 81% after five years, however the statistical basis of this is uncertain.

Table 2. Age- and sex-standardized patient survival, by period and educational group.

	Primary education		Secondary education		Tertiary education	
**Period**	**Patient survival**	**95% Confidence Interval**	**Patient survival**	**95% Confidence Interval**	**Patient survival**	**95% Confidence Interval**
**1 month**	89.2	88.7-89.7	90.7	90.2-91.2	91.5	90.5-92.5
**Year 1**	79.6	79.0-80.2	81.7	81.1-82.3	84.5	83.2-85.6
**Year 2**	73.6	73.0-74.2	76.5	75.9-77.1	80.4	79.1-81.6
**Year 3**	68.5	67.8-69.1	71.7	71.1-72.4	77.0	75.6-78.2
**Year 4**	63.8	63.2-64.5	67.6	67.0-68.3	73.2	71.8-74.5
**Year 5**	59.9	59.2-60.7	64.1	63.4-64.8	69.5	68.0-71.0
**Year 6**	56.3	55.3-57.2	60.5	59.5-61.4	67.5	65.6-69.3

For peer review only - http://bmjopen.bmj.com/site/about/guidelines.xhtml

Table 3. Age- and sex-standardized relative survival, by period and educational group.

	Primary education		Secondary education		Tertiary education	
**Period**	**Relative survival**	**95% Confidence Interval**	**Relative survival**	**95% Confidence Interval**	**Relative survival**	**95% Confidence Interval**
**1 month**	89.6	89.1 - 90.0	91.0	90.5 - 91.5	91.8	90.7 - 92.7
**Year 1**	83.0	82.3 - 83.6	84.6	84.0 - 85.3	87.0	85.6 - 88.2
**Year 2**	79.8	79.0 - 80.5	81.9	81.2 - 82.7	85.2	83.7 - 86.7
**Year 3**	77.1	76.3 - 77.9	79.4	78.5 - 80.2	84.3	82.5 - 85.9
**Year 4**	74.4	73.5 - 75.3	77.2	76.2 - 78.1	82.5	80.4 - 84.3
**Year 5**	72.4	71.3 - 73.5	75.4	74.3 - 76.5	80.8	78.3 - 83.1
**Year 6**	70.2	68.6 - 71.8	73.1	71.4 - 74.7	81.2	77.4 - 84.4

The patterns observed in Table 2 can be explored further by comparing patient survival and expected survival on a continuous time scale (Figure 1). For the primary and secondary education groups, the patient survival curve has a consistently steeper slope than the expected survival curve. In contrast, these curves become nearly parallel in some periods for the tertiary education group.

(Figure 1 about here)

Summarizing the findings from Figure 1 and Table 2, we observed that differences in overall, expected, and relative survival all appeared to increase with time since AMI, to the benefit of patients with higher education levels. The findings of the previously specified sensitivity analyses did not differ meaningfully from the results presented above.

For peer review only

DISCUSSION

The purpose of this study was to compare patient survival with population survival across levels of education, which was used as a marker of socioeconomic status. Our findings confirm previous studies that have documented large and continuing differences in survival (see Osler et al. for a recent overview [12]). Additionally, our analysis demonstrates how these differences in post-AMI survival relate to the expected survival of different educational groups in the general population. According to our results, the relative survival of patients with a primary level of education corresponded to those of patients with a tertiary level of education who are five to ten years older. The main result is that the survival of patients with a tertiary education level remained closest to the survival seen for members of the general population with the same educational background.

A core strength of our study is the use of educational and patient data on the individual level for the entire Norwegian population. This was possible because the Norwegian National Patient Registry was made linkable in 2008, with the tripartite aim of safeguarding access to health care, ensuring appropriate quality of services, and monitoring disease occurrence in the population.
Relative survival has been suggested as a method for monitoring cardiovascular diseases, [14] but few applications have been published [18-22]. Our work appears to be the first study in which expected survival has been adjusted for infarction-related mortality—and this needs to be accounted for when making comparisons. Ideally, patient survival should be viewed in comparison with the expected survival that would be seen in a comparable population that is free of the disease in question. Survival in the general population is often used as an approximate estimate of expected survival, but because deaths among AMI patients constitute a substantial share of the general population's mortality, this approximation can lead to underestimation of expected survival rates [17]. Hence, if we had not adjusted the expected survival rates, our estimates of relative survival would have been higher.

Although we have studied relative survival by education, the same methodology may also facilitate comparisons across countries [30] or over time [21]. Where the comparison is of countries that have different life expectancies or coding practices for causes of death, the use of relative survival provides an external adjustment for survival differences that are unrelated to the disease under study. Therefore,

For peer review only - http://bmjopen.bmj.com/site/about/guidelines.xhtml

relative survival ratios can help to reveal how survival is affected by international differences in disease characteristics and treatments.

A previous, population-based study reported that survival among elderly AMI patients who underwent percutaneous coronary intervention (PCI) was comparable to the general population after about six months, and remained slightly above that of the general population for the remaining three-year follow-up [20]. These estimates of relative survival are higher than our own, which may partly be due to the mentioned difference in the expected survival calculations, but may also be attributable to the fact that PCI patients tend to have higher survival rates than AMI patients, in general.

We have found only two previous studies which have related relative survival to socioeconomic factors [18-19]. For AMI patients who survived the first 24 hours, a Singaporean study reported five-year relative survival ratios of 69%, 73%, and 79%, for Malayan, Chinese, and Indian ethnic groups, respectively [18]. A Spanish study classified AMI patients using a social deprivation index based on census tract [19], but found no association between relative survival and deprivation for men. For women, the association was significant but, surprisingly, patients with intermediate deprivation levels had higher relative survival than the least deprived.

We used attained educational level as a measure of individual socioeconomic status; in part, this measure accounts for the socioeconomic status established in young adulthood [31]. Groups with lower education levels are likely to experience more severe infarctions, owing to for instance more comorbidities and the presence of risk factor accumulation [32]. After an infarction, groups with lower educational attainment will in all likelihood be in command of significantly fewer resources to mobilize [33 34], a situation that affects both clinical outcomes and lifestyle changes [35-37]. Each of these factors is only partially within the direct control of healthcare services. The appropriate channel of influence for these factors also lies with the overall society. All else equal, greater infarction severity and more limited resources may extend the period that patients with low education levels need to recover, thereby leading to reductions in survival that persist for a longer period.

There are some limitations to the study. First is our reliance on at least partially modelled estimates for expected survival. We investigated the plausibility of our results in two sensitivity analyses: One

For peer review only - http://bmjopen.bmj.com/site/about/guidelines.xhtml

sensitivity analysis relied on the latest available estimates from 2010. The other sensitivity analysis used standardized mortality ratios for the years 2008–2010, and applying these ratios to year 2008–2013 life tables from the Human Mortality Database, as suggested by Dickman et al. [16] and implemented by Eleoranta et al. [38]. We did not find any notable differences in our results when we applied these alternative estimates of expected survival.

A second limitation is that we were unable to provide separate corrections of the life tables for infarction-related mortality rates in each of the three education groups. As a sensitivity analysis, we therefore approximated such a correction by setting any difference equal to the relationship for overall cardiovascular mortality [27]. This adjustment is only appropriate to the extent that the relationship between educational level and overall cardiovascular disease is representative of AMI. We believe this to be the case. The application of this sensitivity analysis did not lead to differences from our previously obtained results, perhaps because cardiovascular mortality is responsible for about the same proportion of deaths, even in education groups with quite different rates of overall mortality [12 27 39].

These limitations notwithstanding, we believe that there are clear recommendations to be made on the basis of this study, which may inform models that compare the health benefits and cost consequences of cardiovascular disease interventions [40]. Thus, relevant cost-effectiveness analyses should incorporate the notion of lower relative survival among AMI patients, which extends beyond the first year after AMI (as is common). Further, where appropriate, models should incorporate relationships with received treatment, as in Velders et al. [20].

In our opinion, including expected survival for AMI patients and considering relative survival could provide a complementary estimate of the space in which improvements to patient survival can be made. Regarding our comparisons across levels of socioeconomic status, our findings suggest that both clinically and societally meaningful increases in the number of years lived could be obtained if the relative survival of AMI patients with lower educational levels were increased to the levels observed for patients with higher educational levels.

For peer review only - http://bmjopen.bmj.com/site/about/guidelines.xhtml

Acknowledgements

This study builds upon research infrastructure established by the European Health Care Outcomes, Performance and Efficiency (EuroHOPE) project. We are grateful to the Norwegian Patient Registry and Statistics Norway for allowing us to use their data. We additionally thank Tron Anders Moger and Tor Iversen for commenting on the study.

Contributors

STK conceived the study, managed the data, performed the statistical analyses, and wrote the first draft. KRW participated in the interpretation of the statistical analyses and redrafted the manuscript. Both authors were critically important for the manuscript's intellectual content and have both approved its final version.

Funding

The establishment of the research database was partially funded by the European Union 7th Framework Programme, contract no. 241721,(the EuroHOPE project).

Competing interests

None declared.

Ethics approval

The project has been approved by the Regional Committees for Medical and Health Research Ethics (REC South East Norway), project no. 2009/2114.

Data sharing statement

Due to legal restrictions, we are prevented from sharing individual-level data. Researchers who wish to access data from the Norwegian Directorate of Health (the Norwegian Patient Registry) and Statistics Norway are advised to contact these institutions directly.

REFERENCES

1. Murray CJ, Vos T, Lozano R, *et al.* Disability-adjusted life years (DALYs) for 291 diseases and injuries in 21 regions, 1990–2010: a systematic analysis for the Global Burden of Disease Study 2010. *Lancet* 2012;380(9859):2197–223.
2. Lozano R, Naghavi M, Foreman K, *et al.* Global and regional mortality from 235 causes of death for 20 age groups in 1990 and 2010: a systematic analysis for the Global Burden of Disease Study 2010. *Lancet* 2012;380(9859):2095–128.
3. Strand BH, Tverdal A. Can cardiovascular risk factors and lifestyle explain the educational inequalities in mortality from ischaemic heart disease and from other heart diseases? 26 year follow up of 50,000 Norwegian men and women. *J Epidemiol Community Health* 2004;58(8):705–9.
4. Stringhini S, Dugravot A, Shipley M, *et al.* Health behaviours, socioeconomic status, and mortality: further analyses of the British Whitehall II and the French GAZEL prospective cohorts. *PLoS Med* 2011;8(2):e1000419.

For peer review only - http://bmjopen.bmj.com/site/about/guidelines.xhtml

5. Chaix B, Lindstrom M, Rosvall M, *et al.* Neighbourhood social interactions and risk of acute myocardial infarction. *J Epidemiol Community Health* 2008;62(1):62–8.
6. Barker DJ, Osmond C. Death rates from stroke in England and Wales predicted from past maternal mortality. *Br Med J (Clin Res Ed)* 1987;295(6590):83–6.
7. Barker DJ, Osmond C, Golding J, *et al.* Growth in utero, blood pressure in childhood and adult life, and mortality from cardiovascular disease. *BMJ* 1989;298(6673):564–7.
8. Barker DJ, Winter PD, Osmond C, *et al.* Weight in infancy and death from ischaemic heart disease. *Lancet* 1989;2(8663):577–80.
9. Rajaleid K, Hallqvist J, Koupil I. The effect of early life factors on 28 day case fatality after acute myocardial infarction. *Scand J Public Health* 2009;37(7):720–7.
10. Marmot MG, Rose G, Shipley M, *et al.* Employment grade and coronary heart disease in British civil servants. *J Epidemiol Community Health* 1978;32(4):244–9.
11. Igland J, Vollset SE, Nygard OK, *et al.* Educational inequalities in acute myocardial infarction incidence in Norway: a nationwide cohort study. *PloS One* 2014;9(9):e106898.
12. Osler M, Prescott E, Wium-Andersen IK, *et al.* The impact of comorbid depression on educational inequality in survival after acute coronary syndrome in a cohort of 83 062 patients and a matched reference population. *PloS One* 2015;10(10):e0141598.
13. Mackenbach JP. The persistence of health inequalities in modern welfare states: the explanation of a paradox. *Soc Sci Med* 2012;75(4):761–9.
14. Nelson CP, Lambert PC, Squire IB, *et al.* Relative survival: what can cardiovascular disease learn from cancer? *Eur Heart J* 2008;29(7):941–47.
15. Dickman PW, Adami HO. Interpreting trends in cancer patient survival. *J Intern Med* 2006;260(2):103–17.
16. Dickman PW, Auvinen A, Voutilainen ET, *et al.* Measuring social class differences in cancer patient survival: is it necessary to control for social class differences in general population mortality? A Finnish population-based study. *J Epidemiol Community Health* 1998;52(11):727–34.
17. Talback M, Dickman PW. Estimating expected survival probabilities for relative survival analysis-exploring the impact of including cancer patient mortality in the calculations. *Eur J Cancer* 2011;47(17):2626–32.
18. de Carvalho LP, Gao F, Chen Q, *et al.* Differences in late cardiovascular mortality following acute myocardial infarction in three major Asian ethnic groups. *Eur Heart J Acute Cardiovasc Care* 2014;3(4):354–62.
19. Machon M, Aldasoro E, Martinez-Camblor P, *et al.* Socioeconomic differences in incidence and relative survival after a first acute myocardial infarction in the Basque Country, Spain. *Gac Sanit* 2012;26(1):16–23.
20. Velders MA, James SK, Libungan B, *et al.* Prognosis of elderly patients with ST-elevation myocardial infarction treated with primary percutaneous coronary intervention in 2001 to 2011: a report from the Swedish Coronary Angiography and Angioplasty Registry (SCAAR) registry. *Am Heart J* 2014;167(5):666–73.
21. Alabas OA, Allan V, McLenachan JM, *et al.* Age-dependent improvements in survival after hospitalisation with acute myocardial infarction: an analysis of the Myocardial Ischemia National Audit Project (MINAP). *Age Ageing* 2014;43(6):779–85.
22. Tribouilloy C, Buiciuc O, Rusinaru D, *et al.* Long-term outcome after a first episode of heart failure. A prospective 7-year study. *Int J Cardiol* 2010;140(3):309–14.
23. Igland J, Vollset SE, Nygard OK, *et al.* Educational inequalities in 28 day and 1-year mortality after hospitalisation for incident acute myocardial infarction--a nationwide cohort study. *Int J Cardiol* 2014;177(3):874–80.
24. Kravdal O. A cancer survival model that takes sociodemographic variations in "normal" mortality into account: comparison with other models. *J Epidemiol Community Health* 2002;56(4):309–18.
25. Human Mortality Database. University of California, Berkeley (USA), and Max Planck Institute for Demographic Research (Germany). Available at www.mortality.org (data downloaded on November 10, 2015).

For peer review only - http://bmjopen.bmj.com/site/about/guidelines.xhtml

26. Norwegian Institute of Public Health. Table: [D1: Deaths by sex, age, and detailed cause of death] D1: Dødsfall etter kjønn, alder og detaljert dødsårsak. Available at statistikkbank.fhi.no/dar/ (data downloaded on December 1, 2015).
27. Strand BH, Steingrimsdottir OA, Groholt EK, *et al.* Trends in educational inequalities in cause specific mortality in Norway from 1960 to 2010: a turning point for educational inequalities in cause specific mortality of Norwegian men after the millennium? *BMC Public Health* 2014;14:1208.
28. Dickman PW, Coviello E. Estimating and modeling relative survival. *Stata J* 2015;15(1):186–215.
29. Perme MP, Stare J, Esteve J. On estimation in relative survival. *Biometrics* 2012;68(1):113–20.
30. Coleman MP, Quaresma M, Berrino F, *et al.* Cancer survival in five continents: a worldwide population-based study (CONCORD). *Lancet Oncol* 2008;9(8):730–56.
31. Galobardes B, Shaw M, Lawlor DA, *et al.* Indicators of socioeconomic position (part 1). *J Epidemiol Community Health* 2006;60(1):7–12.
32. Mayer O, Jr., Simon J, Heidrich J, *et al.* Educational level and risk profile of cardiac patients in the EUROASPIRE II substudy. *J Epidemiol Community Health* 2004;58(1):47–52.
33. Cutler DM, Lleras-Muney A. Understanding differences in health behaviors by education. *J Health Econ* 2010;29(1):1–28.
34. Wagstaff A. The demand for health: theory and applications. *J Epidemiol Community Health* 1986;40(1):1–11.
35. Denvir MA, Lee AJ, Rysdale J, *et al.* Influence of socioeconomic status on clinical outcomes and quality of life after percutaneous coronary intervention. *J Epidemiol Community Health* 2006;60(12):1085–88.
36. Alter DA, Iron K, Austin PC, *et al.* Socioeconomic status, service patterns, and perceptions of care among survivors of acute myocardial infarction in Canada. *JAMA* 2004;291(9):1100–7.
37. Chan RH, Gordon NF, Chong A, *et al.* Influence of socioeconomic status on lifestyle behavior modifications among survivors of acute myocardial infarction. *Am J Cardiol* 2008;102(12):1583–8.
38. Eloranta S, Lambert PC, Cavalli-Bjorkman N, *et al.* Does socioeconomic status influence the prospect of cure from colon cancer--a population-based study in Sweden 1965–2000. *Eur J Cancer* 2010;46(16):2965–72.
39. Huisman M, Kunst AE, Bopp M, *et al.* Educational inequalities in cause-specific mortality in middle-aged and older men and women in eight western European populations. *Lancet* 2005;365(9458):493–500.
40. Wisloff T, Selmer RM, Halvorsen S, *et al.* Choice of generic antihypertensive drugs for the primary prevention of cardiovascular disease--a cost-effectiveness analysis. *BMC Cardiovasc Disord* 2012;12:26.

For peer review only

For peer review only - http://bmjopen.bmj.com/site/about/guidelines.xhtml

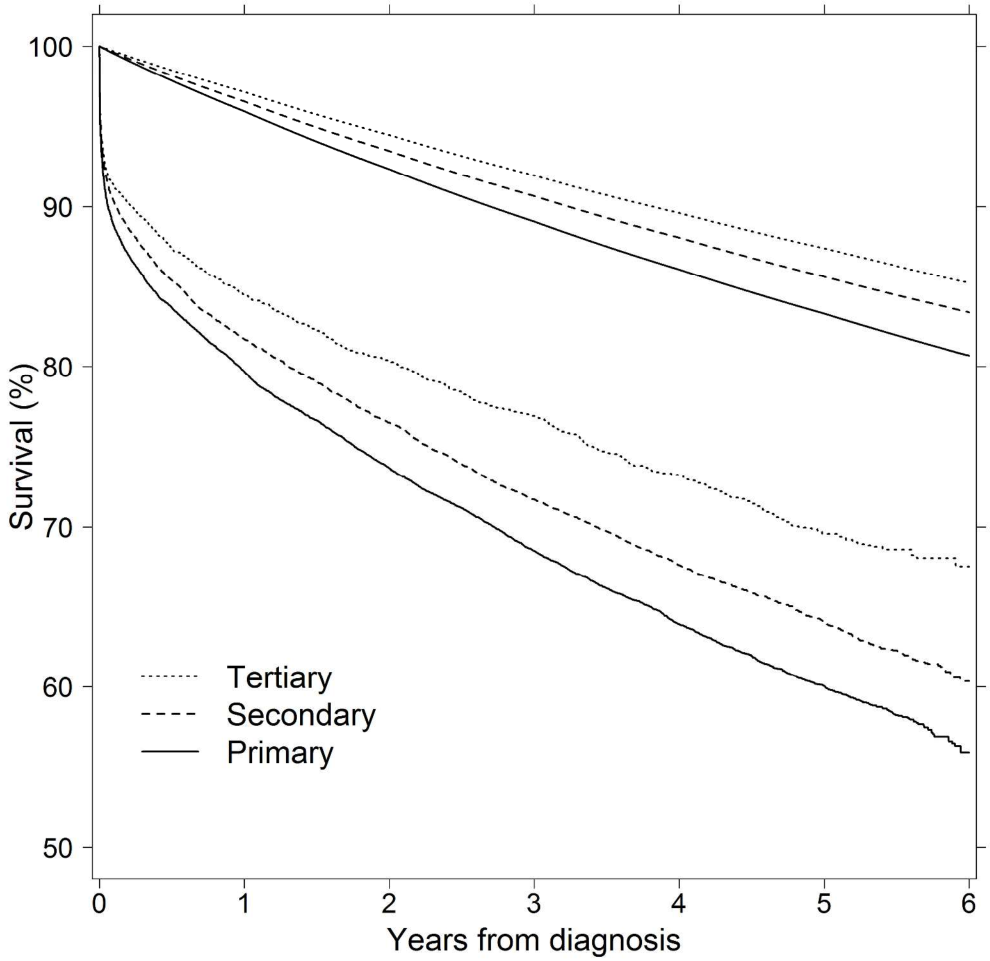

Figure 1. Patient survival and expected survival, according to years since diagnosis and educational group. The upper, nearly linear curves show expected survival rates, as calculated using the Ederer I method. The lower curves show patient survival rates, as estimated using the Kaplan-Meier method. Results are standardized for age and sex.

270x270mm (300 x 300 DPI)

ONLINE SUPPLEMENTARY MATERIAL

S1. Alternative calculation of education-specific expected survival

In our main analysis, we used Poisson regression to predict expected survival for the years 2011–2013. For comparison, we also applied an alternative method, based on an approach of Dickman et al. [1]. In this alternative method, all-cause mortality data obtained from the Human Mortality Database [2] were adjusted based on our data containing educational levels. Let $\mu_{g,t}$ denote the rate of all-cause mortality in year *t* and educational group *g*, and for brevity omit subscripts for age and sex. We then employed the following expression,

$$\mu_{g,t} = \mu_{c,t} \left(\frac{\mu_{g,t^*}}{\mu_{c,t^*}}\right)$$

where values for the whole country have group-subscripts replaced by *c*, and where t^* indicates that estimates for the years 2008–2010 were used. The values $\mu_{c,t}$ were obtained from the Human Mortality Database for each year $t = 2008, \ldots, 2013$, while the standardized mortality ratio $\left(\mu_{g,t^*} / \mu_{c,t^*}\right)$ was taken from our sample for the combined period 2008–2010. The resulting estimates were nearly identical to those in our main analysis.

For peer review only - http://bmjopen.bmj.com/site/about/guidelines.xhtml

S2. Correction for infarction-related mortality and sensitivity analysis

As explained in the main text, expected survival was corrected for mortality related to acute myocardial infarction, using the formula [3]

$$p^* = p^{(1-\alpha)}$$

where p^* is the corrected survival probability, p is the observed survival probability, and α is the proportion of deaths due to acute myocardial infarction. Because the necessary data are unavailable, we were unable to correct for α_g directly. Nonetheless, by emulating the relationship for overall cardiovascular mortality (ICD-10 I00–I99), as represented by λ, we obtained an approximate estimator:

$$\frac{\alpha_{g,t}}{\alpha_{c,t}} \approx \left(\frac{\lambda_{g,t}}{\mu_{g,t}}\right) \Big/ \left(\frac{\lambda_{c,t}}{\mu_{c,t}}\right) \quad \Leftrightarrow \quad \alpha_{g,t} \approx \alpha_{c,t} \left(\frac{\lambda_{g,t^*}}{\lambda_{c,t^*}}\right) \Big/ \left(\frac{\mu_{g,t^*}}{\mu_{c,t^*}}\right)$$

In the rightmost expression we used nationwide mortality data [4] for $\alpha_{c,t}$. The subscript t^* indicates that estimates for 2000–2010 were used for λ and μ [5]. The complex fraction was assumed to equal 1, based on previous findings [5], and varying it over a realistic range only had a minor influence on the results.

REFERENCES FOR THE SUPPLEMENTARY MATERIAL

1. Dickman PW, Auvinen A, Voutilainen ET, *et al.* Measuring social class differences in cancer patient survival: is it necessary to control for social class differences in general population mortality? A Finnish population-based study. *J Epidemiol Community Health* 1998;52(11):727–34.
2. Human Mortality Database. University of California, Berkeley (USA), and Max Planck Institute for Demographic Research (Germany). Available at www.mortality.org (data downloaded on November 10, 2015).
3. Talback M, Dickman PW. Estimating expected survival probabilities for relative survival analysis--exploring the impact of including cancer patient mortality in the calculations. *Eur J Cancer* 2011;47(17):2626–32.
4. Norwegian Institute of Public Health. Table: [D1: Deaths by sex, age, and detailed cause of death] D1: Dødsfall etter kjønn, alder og detaljert dødsårsak. Available at statistikkbank.fhi.no/dar/ (data downloaded on December 1, 2015).
5. Strand BH, Steingrimsdottir OA, Groholt EK, *et al.* Trends in educational inequalities in cause specific mortality in Norway from 1960 to 2010: a turning point for educational inequalities in cause specific mortality of Norwegian men after the millennium? *BMC Public Health* 2014;14:1208.

For peer review only - http://bmjopen.bmj.com/site/about/guidelines.xhtml

STROBE 2007 (v4) Statement—Checklist of items that should be included in reports of *cohort studies*

Section/Topic	Item #	Recommendation	Reported on page #
**Title and abstract**	1	(*a*) Indicate the study's design with a commonly used term in the title or the abstract	Abstract p.2
		(*b*) Provide in the abstract an informative and balanced summary of what was done and what was found	Abstract p.2
**Introduction**			
Background/rationale	2	Explain the scientific background and rationale for the investigation being reported	Introduction p.3
Objectives	3	State specific objectives, including any prespecified hypotheses	Introduction, p.3,
**Methods**			
Study design	4	Present key elements of study design early in the paper	In Abstract, p.2, and Methods, Data, p.5
Setting	5	Describe the setting, locations, and relevant dates, including periods of recruitment, exposure, follow-up, and data collection	Methods, Data, p.5
Participants	6	(*a*) Give the eligibility criteria, and the sources and methods of selection of participants. Describe methods of follow-up	Methods, Data, p.5
		(*b*) For matched studies, give matching criteria and number of exposed and unexposed	Not applicable
Variables	7	Clearly define all outcomes, exposures, predictors, potential confounders, and effect modifiers. Give diagnostic criteria, if applicable	Methods, Data, p.5
Data sources/ measurement	8*	For each variable of interest, give sources of data and details of methods of assessment (measurement). Describe comparability of assessment methods if there is more than one group	Methods, Data, p.5
Bias	9	Describe any efforts to address potential sources of bias	Methods, Statistical analyses, p.5/6
Study size	10	Explain how the study size was arrived at	Methods, Data, p.5
Quantitative variables	11	Explain how quantitative variables were handled in the analyses. If applicable, describe which groupings were chosen and why	Methods, Data, p.5 for education level; Methods, Statistical analyses, p.6/7 for survival outcomes
Statistical methods	12	(*a*) Describe all statistical methods, including those used to control for confounding	Methods, Statistical

			analyses, p. 6/7
		(*b*) Describe any methods used to examine subgroups and interactions	Not applicable
		(*c*) Explain how missing data were addressed	Methods, Data, p.5
		(*d*) If applicable, explain how loss to follow-up was addressed	Methods, Data, p.5
		(*e*) Describe any sensitivity analyses	Methods, Statistical analyses, p.6
**Results**			
Participants	13*	(a) Report numbers of individuals at each stage of study—eg numbers potentially eligible, examined for eligibility, confirmed eligible, included in the study, completing follow-up, and analysed	Methods, Data, p.5
		(b) Give reasons for non-participation at each stage	Methods, Data, p.5
		(c) Consider use of a flow diagram	Not applicable
Descriptive data	14*	(a) Give characteristics of study participants (eg demographic, clinical, social) and information on exposures and potential confounders	Methods, Data, p.5, and Results, Table 1.
		(b) Indicate number of participants with missing data for each variable of interest	Methods, Data, p.5
		(c) Summarise follow-up time (eg, average and total amount)	Methods, Data, p.5
Outcome data	15*	Report numbers of outcome events or summary measures over time	Results, Table 2.
Main results	16	(*a*) Give unadjusted estimates and, if applicable, confounder-adjusted estimates and their precision (eg, 95% confidence interval). Make clear which confounders were adjusted for and why they were included	Results, Table 1.
		(*b*) Report category boundaries when continuous variables were categorized	Methods, Data, p.5, for attained education level
		(*c*) If relevant, consider translating estimates of relative risk into absolute risk for a meaningful time period	Not applicable
Other analyses	17	Report other analyses done—eg analyses of subgroups and interactions, and sensitivity analyses	Results, p.10
**Discussion**			
Key results	18	Summarise key results with reference to study objectives	Discussion, p.10
**Limitations**			
Interpretation	20	Give a cautious overall interpretation of results considering objectives, limitations, multiplicity of analyses, results from similar studies, and other relevant evidence	p.11/12
Generalisability	21	Discuss the generalisability (external validity) of the study results	p.11
**Other information**			

For peer review only

Funding	22	Give the source of funding and the role of the funders for the present study and, if applicable, for the original study on which the present article is based	p.14

*Give information separately for cases and controls in case-control studies and, if applicable, for exposed and unexposed groups in cohort and cross-sectional studies.

Note: An Explanation and Elaboration article discusses each checklist item and gives methodological background and published examples of transparent reporting. The STROBE checklist is best used in conjunction with this article (freely available on the Web sites of PLoS Medicine at http://www.plosmedicine.org/, Annals of Internal Medicine at http://www.annals.org/, and Epidemiology at http://www.epidem.com/). Information on the STROBE Initiative is available at www.strobe-statement.org.

For peer review only

BMJ Open

Educational attainment and differences in relative survival after acute myocardial infarction in Norway: a registry-based population study

Journal:	*BMJ Open*
Manuscript ID	bmjopen-2016-014787.R1
Article Type:	Research
Date Submitted by the Author:	14-Jun-2017
Complete List of Authors:	Klitkou, Søren; University of Oslo, Health Management and Health Economics<br>Wangen, Knut ; University of Oslo, Health Management and Health Economics
**Primary Subject Heading**:	Public health
Secondary Subject Heading:	Cardiovascular medicine
Keywords:	Coronary heart disease < CARDIOLOGY, Record linkage, Health inequalities, Education, Mortality

SCHOLARONE™
Manuscripts

Title:

Educational attainment and differences in relative survival after acute myocardial infarction in Norway: a registry-based population study

Søren T. Klitkou[1], Knut R. Wangen[1]

[1] Department of Health Management and Health Economics, Institute of Health and Society, University of Oslo, Oslo, Norway

Corresponding author:

Søren Toksvig Klitkou

Department of Health Management and Health Economics

University of Oslo

Postal address: Postbox 1089 Blindern, NO - 0317 Oslo, Norway

Phone number: +47 228 45 028

Fax number: +47 228 45 091

Email address: s.t.klitkou@medisin.uio.no

Key words: Coronary heart disease, Record linkage, Health inequalities, Education, Mortality

Word count excluding Tables, Abstract, Strengths and limitations of this study, What is known on this topic, What this study adds, Acknowledgments, References, and Supplementary files: 3208

Number of tables = 3; Figures = 1; References = 40

Article type: observational study (research report)

Abstract

For peer review only

Background: Although there is a broad societal interest in socioeconomic differences in survival after an acute myocardial infarction, only a few studies have investigated how such differences relate to the survival in general population groups. We aimed to investigate education-specific survival after acute myocardial infarction and to compare this with the survival of corresponding groups in the general population.

Methods: Our study included the entire population of Norwegian patients admitted to hospitals for acute myocardial infarction during 2008–2010, with a six-year follow-up period. Patient survival was measured relative to the expected survival in the general population for three educational groups: primary, secondary, and tertiary. Education-, sex-, age-, and calendar-year-specific expected survival were obtained from population life tables and adjusted for the presence of infarction-related mortality.

Results: Six-year patient survivals were, respectively, 56.3% (55.3-57.2) and 65.5% (65.6-69.3) for the primary and tertiary educational groups (95% confidence intervals). Also six-year relative survival was markedly lower for the primary educational group: 70.2% (68.6 - 71.8) versus 81.2% (77.4 - 84.4). Throughout the follow-up period, patient survival tended to remain lower than the survival in the general population with the same educational background.

Conclusion: Both patient survival and relative survival after acute myocardial infarction are positively associated with educational level. Our findings may suggest that secondary prevention has been more effective for the highly educated.

For peer review only

For peer review only - http://bmjopen.bmj.com/site/about/guidelines.xhtml

Strengths and limitations of this study

- The relative survival framework made possible the comparison of patient survival to population survival for a national cohort of AMI patients and to distinguish how groups defined by educational level fared compared to their group in the population.
- The use of nationwide registry data from patients and the general population give findings that are representative for Norway for the years of diagnosis from 2008 to 2010 with a maximum follow-up of six years until the end of 2013.
- The study has low levels of missing data on education (<2%) and identical definitions of educational attainment in patient and population groups.
- It is not possible from the study to attribute causality to the age- and sex-standardized differences between educational groups regarding patient survival, the expected survival in the general population, and the relative survival of AMI patients.

For peer review only - http://bmjopen.bmj.com/site/about/guidelines.xhtml

INTRODUCTION

Socioeconomic differences in survival after acute myocardial infarction (AMI) are of broad societal interest, especially because ischemic heart disease is the single most important cause of death, life-years lost prematurely, and life-years lost to disability.[1,2] The causes of heart disease and AMI continue to be associated with lifestyles [3 4]; and life conditions,[5-9] resulting in substantial socioeconomic differences in the rates of disease onset and mortality, which correlate with measures such as education level, income, and occupational class.[10-12] Inequalities in mortality have widened in both absolute and relative terms, a public health phenomenon that has been described as a great disappointment in highly developed welfare states.[13] This widening inequality is in large part attributable to the persistent relationship between socioeconomic status and mortality from cardiovascular disease.

Patient survival following AMI is commonly assessed using overall patient survival.[14] This can be appropriate from a clinical perspecitve, say when comparing treatment strategies. However, AMI occurs more frequently in age groups with elevated background mortality and it is therefore of interest to measure patient survial relative to the survival of the general population. In relative survival this comparison is accomplished by life-tables for population survival.[15]

Dickman et al. emphasized the importance of stratifying the general population life-tables according to the adopted measure of socioeconomic status when the aim is to compare separate social groups.[16] The relative survival approach was later refined to situations where the disease of interest had a substantial influence on population survival – typically when a relatively common disease causes high mortality.[17] The principle of the correction was to adjust the population survival so resembling survival in a population *without* the disease of interest.

Socioeconomic and demographic gradients in relative survival have been studied previously for AMI patients. A Singaporean study found lower relative survival among the Malayan minority, as compared with the Chinese and Indian ethnic groups,[18] while a Spanish study reported no association between relative survival and a social deprivation index,[19] However, neither these nor similar studies of cardiovascular disease applied the mentioned refined approach.[18-22]

For peer review only - http://bmjopen.bmj.com/site/about/guidelines.xhtml

In the context of this prior research, our aim was to investigate relative survival ratios after AMI for patients with different educational backgrounds in Norway. We used registry data that covered the entire Norwegian population. A single-payer healthcare system and a tuition-free higher education system are provided by the public sector in Norway. In principle, this offers the whole population equal access to healthcare and education. Educational inequalities in 28-day and 1-year AMI survival have been reported previously,[23] but no comparison with the general population has been published to date. Additionally, in contrast to previous studies on relative survival for cardiovascular disease,[18-22] we have accounted for AMI mortality in the general population.

METHODS

Data

For the current study, we considered all hospital admissions for AMI (ICD-10 I21or I22 as main diagnosis) that were included in the Norwegian National Patient Registry for the period 2008–2010. Information on patient survival was derived from date of death, from the Causes of Death Registry for the period 2008–2013. This registry covers all deaths in Norway and deaths of Norwegians who die abroad. Data on educational level were obtained from the National Database on Education.

We identified 35,045 patients aged 40–94 years with an AMI diagnosis in the patient registry. Patients who were younger or older at the time of infarction were excluded because of small numbers of patients in these groups. For patients with multiple AMI episodes during the observation period (2008-2010), only the first episode was included in the analysis. Information on AMI episodes prior to the observation period were not available. Educational level was defined as the highest completed level of education, and was categorized as follows: primary (6–9 years of schooling), secondary (10–12 years of schooling) or tertiary (university or college degree). We excluded 637 patients with missing information on attained education, leaving a total sample of 34,408 patients. In all 12,135 persons died during the course of follow-up. The observed number of person-years was 116,269, with an average follow-up of 3.4 years per person.

For peer review only - http://bmjopen.bmj.com/site/about/guidelines.xhtml

To calculate relative survival ratios, we obtained life tables from Statistics Norway for the calendar years 1999–2010, which included data by educational level, sex, and 1-year age interval. The life tables included all deaths and person-years, permitting the calculation of expected survival. Importantly for our application, the patients and the general population were stratified using identical definitions of educational groups.[16,24]

Statistical analyses

For a given calendar year and population stratum (educational level, sex, and age), relative survival was defined as the ratio of survival among the AMI patients to the expected survival for the general population.

The expected survival was estimated in two steps: In the first step, overall expected survival for the years 2008–2010 was obtained directly from the life tables covering the years 1999–2010. Data for the years 2011-2013 were not available and where thus extrapolated based on the 1999-2010 data. For this purpose we estimated six Poisson regression models – one for each combination of educational level and sex. The dependent variable was the number of deaths per person-year, observed for each one-year age category (40-94 years) and for each calendar year (1999-2010). The independent variables included a linear trend for calendar year and restricted cubic splines to represent a possible non-linear age effect,[25] where the number of knots used was decided by Akaike Information Criterion. The estimated models were then used to predict values for 2011-2013. As a sensitivity analysis, we used two alternative approaches to obtain values for 2011-2013: One approach, suggested by Dickman et al. and explained in supplementary description S1,[16] used standardized mortality ratios by educational level for the years 2008–2010 to adjust Norwegian mortality ratios available from the Human Mortality Database for the years 2008-2013.[26] A second approach consisted of simply forwarding the estimates from the last available year of our life table data (i.e., 2010). In the second step, we adjusted the overall expected survival for the substantial proportion of deaths due to AMI – by using Talbäck and Dickman's correction formula.[17] To this end, we obtained data from the Norwegian Institute of Public Health on the sex and age-group specific proportions of deaths due to AMI.[27] As these data did not include educational level, we performed a sensitivity analysis based on published comparisons of

For peer review only - http://bmjopen.bmj.com/site/about/guidelines.xhtml

cardiovascular mortality (ICD-10 I00–I99) with overall mortality (see supplementary description S2 for further details).[28]

We summarized survival differences by level of education at the end of follow-up, as stratified by sex and 5-year age groups. To investigate the time course of survival since AMI, we calculated survival for the first month and each year thereafter. For each of these time points, we calculated relative survival (using the Ederer II method) by directly standardizing the three educational groups to the overall age and sex distribution.[25,29] The standardized survival was defined as the weighted average of the survival in each age and sex group, where the weights equalled the proportions of patients in each group. Finally, we performed a continuous-time analysis of survival, in which the time course of survival since AMI was estimated using the Kaplan-Meier and the time course of expected survival was estimated using the Ederer I method,[25] as standardized by reweighting to the overall age and sex distribution. Stata 14 (StataCorp, College Station, TX, USA) and the Strs-procedure were used for the calculations in the tables.[25] Additionally, we used R version 3.1.2 and the Survival package for continuous-time estimates.

RESULTS

The average age at hospitalization was 71 years, and 36% of the patients were female. Of the 34,408 patients, 14,360 had completed primary education, 15,527 had completed secondary education, and 4,521 had completed tertiary education.

Table 1 shows the survival estimates, stratified by sex, education, and 5-year age groups: patient survival at six years of follow-up; the expected survival if the patients had experienced the mortality rates of the general population; and the relative survival, defined as the ratio of the patient survival to the expected survival. The expected survival is obtained from the full population and has a clear, consistent pattern: for a given sex and educational group, it is decreasing in age; for a given age and educational level, it is higher for females than for males, and; for a given age and sex, it is increasing by educational level. Patient survival and relative survival also vary but their patterns appear to be more consistent for males (n=21,878) than for females (n=12,530), presumably due to the higher

For peer review only - http://bmjopen.bmj.com/site/about/guidelines.xhtml

number of male patients. For males above 55 years, patient survival clearly differs according to educational level. For example, there is a notable difference for the age group 55–59 years, where the respective patient survivals in the primary, secondary, and tertiary educational groups were 80.9%, 87.1%, and 92.5%. Dividing by the corresponding expected survivals, this translates into the relative survivals of 85.8%, 90.1% and 94.4%, for the primary, secondary, and tertiary educational groups, respectively. A higher relative survival indicates that the patient survival remained closer to the survival among members of the general population background. For females, the relative survival also has a similar socioeconomic gradient, except for the age groups 50-54, 55-59, and 80-84, but the 95% confidence intervals are rather wide.

For peer review only - http://bmjopen.bmj.com/site/about/guidelines.xhtml

Table 1. The 6-year survival rates of patients after acute myocardial infarction, according to sex, age, and education level. Observed patient survival, expected survival,[1] and relative survival ratios are shown.

	Males											
	Primary education				Secondary education				Tertiary education			
Age, years	N	PS (95% CI)	ES	RS (95% CI)	N	PS (95% CI)	ES	RS (95% CI)	N	PS (95% CI)	ES	RS (95% CI)
40-49	702	90.0 (86.5-92.6)	98.2	91.7 (88.1-94.4)	814	93.6 (90.8-95.6)	99.0	94.5 (91.7-96.5)	316	89.2 (83.4-93.1)	99.5	89.7 (83.9-93.6)
50-54	531	88.0 (83.6-91.3)	96.4	91.2 (86.7-94.6)	985	89.2 (86.3-91.6)	98.0	91.1 (88.1-93.5)	317	91.5 (86.1-94.9)	98.9	92.6 (87.1-96.0)
55-59	615	80.9 (76.3-84.7)	94.2	85.8 (81.0-89.9)	1310	87.1 (84.4-89.4)	96.7	90.1 (87.3-92.5)	461	92.5 (88.3-95.2)	98.0	94.4 (90.2-97.2)
60-64	819	74.6 (70.4-78.3)	92.0	81.1 (76.6-85.1)	1765	79.9 (77.3-82.3)	94.5	84.6 (81.8-87.1)	583	86.3 (81.9-89.6)	96.7	89.2 (84.7-92.7)
65-69	794	63.2 (58.8-67.3)	87.9	71.9 (66.9-76.6)	1294	72.7 (69.3-75.7)	91.5	79.4 (75.8-82.8)	495	81.7 (76.5-85.8)	94.2	86.7 (81.3-91.1)
70-74	870	54.5 (50.3-58.5)	82.5	66.1 (61.0-71.0)	1148	59.7 (56.0-63.2)	86.6	68.9 (64.7-73.0)	366	67.9 (61.3-73.6)	90.7	74.9 (67.5-81.2)
75-79	1020	40.4 (36.8-44.0)	72.4	55.8 (50.9-60.7)	1108	43.4 (39.9-46.9)	76.5	56.8 (52.2-61.3)	319	54.9 (47.8-61.4)	81.6	67.3 (58.6-75.2)
80-84	1124	23.9 (21.1-26.7)	56.2	42.4 (37.6-47.4)	1096	27.1 (24.2-30.1)	62.4	43.4 (38.7-48.2)	299	31.1 (25.2-37.2)	68.6	45.4 (36.8-54.3)
85-89	846	11.4 (9.3-13.8)	38.5	29.6 (24.1-35.8)	863	12.7 (10.5-15.2)	42.3	30.1 (24.8-36.0)	232	24.4 (18.5-30.8)	50.0	48.8 (37.0-61.5)
90-94	363	5.1 (3.1-7.7)	21.4	23.7 (14.5-36.2)	345	6.0 (3.8-8.9)	22.8	26.3 (16.5-39.2)	77	13.2 (6.5-22.4)	28.5	46.4 (22.8-78.5)

For peer review only

For peer review only - http://bmjopen.bmj.com/site/about/guidelines.xhtml

Age, years	Females											
	Primary education				Secondary education				Tertiary education			
	N	PS (95% CI)	ES	RS (95% CI)	N	PS (95% CI)	ES	RS (95% CI)	N	PS (95% CI)	ES	RS (95% CI)
40-49	170	83.8 (74.6-89.9)	98.9	84.8 (75.4-90.9)	152	88.8 (79.6-94.0)	99.4	89.4 (80.1-94.6)	80	90.5 (76.6-96.3)	99.6	90.9 (76.9-96.7)
50-54	162	90.6 (82.1-95.2)	97.7	92.8 (84.0-97.5)	176	84.3 (75.3-90.2)	98.6	85.5 (76.3-91.5)	83	88.6 (74.8-95.1)	98.9	89.6 (75.7-96.2)
55-59	177	89.3 (81.0-94.1)	96.6	92.4 (83.9-97.4)	311	89.1 (83.2-93.0)	97.9	90.9 (85.0-94.9)	89	97.8 (85.3-99.7)	98.6	99.2 (86.5-101.1)
60-64	360	69.8 (63.1-75.5)	95.0	73.5 (66.5-79.5)	434	79.7 (74.1-84.3)	96.5	82.6 (76.7-87.3)	121	86.2 (75.1-92.5)	97.8	88.1 (76.8-94.6)
65-69	427	65.2 (59.0-70.6)	92.6	70.4 (63.8-76.3)	418	70.6 (64.5-75.9)	95.0	74.3 (67.9-79.9)	102	87.2 (74.9-93.7)	96.5	90.3 (77.7-97.1)
70-74	644	55.0 (50.1-59.6)	88.9	61.9 (56.3-67.1)	501	61.6 (56.0-66.8)	92.4	66.7 (60.6-72.3)	108	67.4 (54.6-77.4)	94.1	71.7 (58.0-82.3)
75-79	942	41.1 (37.4-44.8)	82.1	50.1 (45.5-54.6)	577	48.5 (43.5-53.4)	86.2	56.3 (50.4-61.9)	119	64.1 (52.0-74.0)	89.1	72.0 (58.4-83.0)
80-84	1376	24.5 (22.0-27.0)	68.9	35.5 (31.9-39.2)	834	31.3 (27.8-35.0)	74.3	42.2 (37.4-47.1)	125	32.3 (23.1-41.8)	78.6	41.1 (29.4-53.2)
85-89	1548	13.8 (12.1-15.7)	51.2	27.0 (23.5-30.7)	864	18.4 (15.6-21.3)	56.7	32.4 (27.6-37.5)	146	22.7 (15.7-30.5)	59.7	38.0 (26.2-51.2)
90-94	870	8.4 (6.6-10.5)	31.0	27.1 (21.3-33.7)	531	9.7 (7.3-12.6)	34.5	28.1 (21.1-36.3)	83	14.5 (7.6-23.5)	41.1	35.2 (18.4-57.3)

[1] Expected survival has been corrected for mortality from acute myocardial infarction. Measurement errors in expected survival were ignored. PS: patient survival, ES: expected survival, RS: relative survival. The patient survival estimates are by year six and were based on the subsample of patients diagnosed with AMI in 2008 with complete follow-up.

For peer review only

For peer review only - http://bmjopen.bmj.com/site/about/guidelines.xhtml

The results in Tables 2 and 3 are standardized to the combined age- and sex distribution of all the observed patients. Table 2 presents the time course of patient survival. The six-year survival was 56.3% and 67.5 % for patients with primary and tertiary education, respectively. Accounting for the expected survival in the general population, Table 3 presents the time course of relative survival ratios over the follow-up period. In each year, the AMI patients fared worse than their counterparts in the general population. Survival decreased substantially during the first year since AMI, for which the relative survival ratios were 83.0%, 84.6%, and 87.0% in the primary, secondary, and tertiary education groups, respectively. By year six, ratios of relative survival were 70.2%, 73.1%, and 81.2% in these groups—the difference in relative survival had become markedly larger. For the primary and secondary education groups, the decline in relative survival was monotonic over the six-year period. In contrast, for the tertiary education group, the relative survival ratio appeared to stabilize at approximately 81% after five years, however the statistical basis of this is uncertain.

Table 2. Age- and sex-standardized patient survival, by period and educational group (N=34,408).

	Primary education		Secondary education		Tertiary education	
**Period**	**Patient survival**	**95% Confidence Interval**	**Patient survival**	**95% Confidence Interval**	**Patient survival**	**95% Confidence Interval**
**1 month**	89.2	88.7-89.7	90.7	90.2-91.2	91.5	90.5-92.5
**Year 1**	79.6	79.0-80.2	81.7	81.1-82.3	84.5	83.2-85.6
**Year 2**	73.6	73.0-74.2	76.5	75.9-77.1	80.4	79.1-81.6
**Year 3**	68.5	67.8-69.1	71.7	71.1-72.4	77.0	75.6-78.2
**Year 4**	63.8	63.2-64.5	67.6	67.0-68.3	73.2	71.8-74.5
**Year 5**	59.9	59.2-60.7	64.1	63.4-64.8	69.5	68.0-71.0
**Year 6**	56.3	55.3-57.2	60.5	59.5-61.4	67.5	65.6-69.3

For peer review only - http://bmjopen.bmj.com/site/about/guidelines.xhtml

Table 3. Age- and sex-standardized relative survival, by period and educational group (N=34,408).

	Primary education		Secondary education		Tertiary education	
Period	Relative survival	95% Confidence Interval	Relative survival	95% Confidence Interval	Relative survival	95% Confidence Interval
**1 month**	89.6	89.1 - 90.0	91.0	90.5 - 91.5	91.8	90.7 - 92.7
**Year 1**	83.0	82.3 - 83.6	84.6	84.0 - 85.3	87.0	85.6 - 88.2
**Year 2**	79.8	79.0 - 80.5	81.9	81.2 - 82.7	85.2	83.7 - 86.7
**Year 3**	77.1	76.3 - 77.9	79.4	78.5 - 80.2	84.3	82.5 - 85.9
**Year 4**	74.4	73.5 - 75.3	77.2	76.2 - 78.1	82.5	80.4 - 84.3
**Year 5**	72.4	71.3 - 73.5	75.4	74.3 - 76.5	80.8	78.3 - 83.1
**Year 6**	70.2	68.6 - 71.8	73.1	71.4 - 74.7	81.2	77.4 - 84.4

The patterns observed in Table 2 can be explored further by comparing patient survival and expected survival on a continuous time scale (Figure 1). For the primary and secondary education groups, the patient survival curve has a consistently steeper slope than the expected survival curve. In contrast, these curves become nearly parallel in some periods for the tertiary education group.

(Figure 1 about here)

Summarizing the findings from Figure 1 and Table 2, we observed that differences in overall, expected, and relative survival all appeared to increase with time since AMI, to the benefit of patients with higher education levels. The findings of the previously specified sensitivity analyses did not differ meaningfully from the results presented above.

DISCUSSION

For peer review only

The purpose of this study was to compare patient survival with population survival across levels of education, which was used as a marker of socioeconomic status. Our findings confirm previous studies that have documented large and continuing differences in patient survival, see Osler et al. for a recent overview.[12] Additionally, our analysis demonstrates how these differences in post-AMI survival relate to the expected survival of different educational groups in the general population. According to our results, the relative survival of patients with a primary level of education corresponded to those of patients with a tertiary level of education who are five to ten years older. The main result is that the survival of patients with a tertiary education level remained closest to the survival seen for members of the general population with the same educational background. Given the relation of socioeconomic status to general population mortality, [4 13] we expect that the current result would be replicable in other countries and for other diseases exhibiting a socioeconomic dimension to survival.

A core strength of our study is the use of educational and patient data on the individual level for the entire Norwegian population. This was possible because the Norwegian National Patient Registry was made linkable in 2008, with the tripartite aim of safeguarding access to health care, ensuring appropriate quality of services, and monitoring disease occurrence in the population.

Relative survival has been suggested as a method for monitoring cardiovascular diseases,[14] but few applications have been published.[18-22] Our work appears to be the first study in which expected survival has been adjusted for infarction-related mortality—and this needs to be accounted for when making comparisons. Ideally, patient survival should be viewed in comparison with the expected survival that would be seen in a comparable population that is free of the disease in question. Survival in the general population is often used as an approximate estimate of expected survival, but because deaths among AMI patients constitute a substantial share of the general population's mortality, this approximation can lead to underestimation of expected survival.[17] Hence, if we had not adjusted the expected survival, our estimates of relative survival would have been higher.

Although we have studied relative survival by education, the same methodology may also facilitate comparisons across countries or over time.[21,30] Where the comparison is of countries that have different life expectancies or coding practices for causes of death, the use of relative survival provides

For peer review only - http://bmjopen.bmj.com/site/about/guidelines.xhtml

an external adjustment for survival differences that are unrelated to the disease under study. Therefore, relative survival ratios can help to reveal how survival is affected by international differences in disease characteristics and treatments.

A previous, population-based study reported that survival among elderly AMI patients who underwent percutaneous coronary intervention (PCI) was comparable to the general population after about six months, and remained slightly above that of the general population for the remaining three-year follow-up.[20] These estimates of relative survival are higher than our own, which may partly be due to the mentioned difference in the expected survival calculations, but may also be attributable to the fact that PCI patients tend to have higher survival than AMI patients, in general.

We have found only two previous studies which have related relative survival of AMI patients to socioeconomic factors.[18,19] For AMI patients who survived the first 24 hours, a Singaporean study reported five-year relative survival ratios of 69%, 73%, and 79%, for Malayan, Chinese, and Indian ethnic groups, respectively.[18] A Spanish study classified AMI patients using a social deprivation index based on census tract,[19] but found no association between relative survival and deprivation for men. For women, the association was significant but, surprisingly, patients with intermediate deprivation levels had higher relative survival than the least deprived.

We used attained educational level as a measure of individual socioeconomic status. The educational level is usually attained in young adulthood when populations are overall in good health, and determined before later life social aspects such as income or occupation.[31] Groups with lower education levels are likely to experience more severe infarctions, owing to for instance more comorbidities and the presence of risk factor accumulation.[32] After an infarction, groups with lower educational attainment will in all likelihood be in command of significantly fewer resources to mobilize,[33,34] a situation that affects both clinical outcomes and lifestyle changes.[35-37] Each of these factors is only partially within the direct control of healthcare services, whose mandate is to base choice of treatment on the existing health and comorbidities of the patient. The appropriate channel of influence for factors affecting survival therefore also lies with the overall society. All else equal, greater infarction severity and more limited resources may therefore extend the period that patients

For peer review only - http://bmjopen.bmj.com/site/about/guidelines.xhtml

For peer review only

with low education levels need to recover, thereby leading to reductions in survival that persist for a longer period. Similar mechanisms could apply for other causes of mortality – such as strokes, some types of cancers, and accidents– which may explain the educational differences in expected survival.[28] Also, both patient and relative survival had clear educational differences in the aggregate (Tables 2 and 3); while the differences were less clear for some, particularly younger age groups (Table 1). It is beyond our scope to investigate this further, but it could be due to factors not accounted for – such as differences by birth cohorts in the advantage education confers [31]; or due to the relatively few observations in the disaggregated groups.

There are some limitations to the study. First, our data do not contain information on AMI episodes prior to the observation period (2008-2010). Thus, our measurements of patient survival were obtained from a group where some patients had their first AMI episode while others may have had two or more. Previous studies that have reported survival after the first AMI episode are relevant for comparison.[12] However, it should be noted that if patient survival is generally higher after the first AMI episode than after later AMI episodes, our results would tend to be lower than for a sample with first AMI episode patients only. This effect of mixing patients may not have uniform size across educational groups, partly because educational differences affect the incidence of AMI and because their survival after an AMI differ, as demonstrated by our results.[11]

The second limitation is our reliance on at least partially modelled estimates for expected survival. We investigated the plausibility of our results in two sensitivity analyses: One sensitivity analysis relied on the latest available estimates from 2010. The other sensitivity analysis used standardized mortality ratios for the years 2008–2010, and applying these ratios to year 2008–2013 life tables from the Human Mortality Database, as suggested by Dickman et al.[16] and implemented by Eleoranta et al. [38]. We did not find any notable differences in our results when we applied these alternative estimates of expected survival.

A third limitation is that we were unable to provide separate corrections of the life tables for infarction-related mortality in each of the three education groups. As a sensitivity analysis, we therefore approximated such a correction by setting any difference equal to the relationship for overall

For peer review only - http://bmjopen.bmj.com/site/about/guidelines.xhtml

cardiovascular mortality.[28] This adjustment is only appropriate to the extent that the relationship between educational level and overall cardiovascular disease is representative of AMI. We believe this to be the case. The application of this sensitivity analysis did not lead to differences from our previously obtained results, perhaps because cardiovascular mortality is responsible for about the same proportion of deaths, even in education groups with quite different overall mortality.[12,28,39]

These limitations notwithstanding, we believe that there are clear recommendations to be made on the basis of this study, which may inform models that compare the health benefits and cost consequences of cardiovascular disease interventions.[40] Thus, relevant cost-effectiveness analyses should incorporate the notion of lower relative survival among AMI patients, which extends beyond the first year after AMI (as is common). Further, where appropriate, models should incorporate relationships with received treatment, as in Velders et al. [20].

In our opinion, including expected survival for AMI patients and considering relative survival could provide a complementary estimate of the space in which improvements to patient survival can be made. Regarding our comparisons across levels of socioeconomic status, our findings suggest that both clinically and societally meaningful increases in the number of years lived could be obtained if the relative survival of AMI patients with lower educational levels were increased to the levels observed for patients with higher educational levels.

For peer review only

For peer review only - http://bmjopen.bmj.com/site/about/guidelines.xhtml

Acknowledgements

This study builds upon research infrastructure established by the European Health Care Outcomes, Performance and Efficiency (EuroHOPE) project. We are grateful to the Norwegian Patient Registry and Statistics Norway for allowing us to use their data. We thank Tron Anders Moger and Tor Iversen for commenting on the study. We additionally thank the reviewers for providing constructive comments that helped clarify and improve the paper.

Contributors

STK conceived the study, managed the data, performed the statistical analyses, and wrote the first draft. KRW participated in the interpretation of the statistical analyses and in redrafting the manuscript. Both authors were critically important for the manuscript's intellectual content and have both approved its final version.

Funding

The establishment of the research database was partially funded by the European Union 7th Framework Programme, contract no. 241721,(the EuroHOPE project).

Competing interests

None declared.

Ethics approval

The project has been approved by the Regional Committees for Medical and Health Research Ethics (REC South East Norway), project no. 2009/2114.

Data sharing statement

Due to legal restrictions we are prevented from sharing individual level data. Researchers who wish to access data from the Norwegian Directorate of Health (the Norwegian Patient Registry) and Statistics Norway are advised to contact these institutions directly.

REFERENCES

1. Murray CJ, Vos T, Lozano R, *et al.* Disability-adjusted life years (DALYs) for 291 diseases and injuries in 21 regions, 1990–2010: a systematic analysis for the Global Burden of Disease Study 2010. *Lancet* 2012;380:2197–223.
2. Lozano R, Naghavi M, Foreman K, *et al.* Global and regional mortality from 235 causes of death for 20 age groups in 1990 and 2010: a systematic analysis for the Global Burden of Disease Study 2010. *Lancet* 2012;380:2095–128.
3. Strand BH, Tverdal A. Can cardiovascular risk factors and lifestyle explain the educational inequalities in mortality from ischaemic heart disease and from other heart diseases? 26 year follow up of 50,000 Norwegian men and women. *J Epidemiol Community Health* 2004;58:705–9.

For peer review only - http://bmjopen.bmj.com/site/about/guidelines.xhtml

For peer review only

4. Stringhini S, Dugravot A, Shipley M, *et al.* Health behaviours, socioeconomic status, and mortality: further analyses of the British Whitehall II and the French GAZEL prospective cohorts. *PLoS Med* 2011;8:e1000419.
5. Chaix B, Lindstrom M, Rosvall M, Merlo J. Neighbourhood social interactions and risk of acute myocardial infarction. *J Epidemiol Community Health* 2008;62:62–8.
6. Barker DJ, Osmond C. Death rates from stroke in England and Wales predicted from past maternal mortality. *Br Med J (Clin Res Ed)* 1987;295:83–6.
7. Barker DJ, Osmond C, Golding J, Kuh D, Wadsworth ME. Growth in utero, blood pressure in childhood and adult life, and mortality from cardiovascular disease. *BMJ* 1989;298:564–7.
8. Barker DJP, Winter PD, Osmond C, Margetts B, Simmonds SJ. Weight in infancy and death from ischaemic heart disease. *Lancet* 1989;2:577–80.
9. Rajaleid K, Hallqvist J, Koupil I. The effect of early life factors on 28 day case fatality after acute myocardial infarction. *Scand J Public Health* 2009;37:720–7.
10. Marmot MG, Rose G, Shipley M, Hamilton PJ. Employment grade and coronary heart disease in British civil servants. *J Epidemiol Community Health* 1978;32:244–9.
11. Igland J, Vollset SE, Nygard OK, Sulo G, Ebbing M, Tell GS. Educational inequalities in acute myocardial infarction incidence in Norway: a nationwide cohort study. *PloS One* 2014;9:e106898.
12. Osler M, Prescott E, Wium-Andersen IK, *et al.* The impact of comorbid depression on educational inequality in survival after acute coronary syndrome in a cohort of 83 062 patients and a matched reference population. *PloS One* 2015;10:e0141598.
13. Mackenbach JP. The persistence of health inequalities in modern welfare states: the explanation of a paradox. *Soc Sci Med* 2012;75:761–9.
14. Nelson CP, Lambert PC, Squire IB, Jones DR. Relative survival: what can cardiovascular disease learn from cancer? *Eur Heart J* 2008;29:941–47.
15. Dickman PW, Adami HO. Interpreting trends in cancer patient survival. *J Intern Med* 2006;260:103–17.
16. Dickman PW, Auvinen A, Voutilainen ET, Hakulinen T. Measuring social class differences in cancer patient survival: is it necessary to control for social class differences in general population mortality? A Finnish population-based study. *J Epidemiol Community Health* 1998;52:727–34.
17. Talback M, Dickman PW. Estimating expected survival probabilities for relative survival analysis–exploring the impact of including cancer patient mortality in the calculations. *Eur J Cancer* 2011;47:2626–32.
18. De Carvalho LP, Gao F, Chen Q, *et al.* Differences in late cardiovascular mortality following acute myocardial infarction in three major Asian ethnic groups. *Eur Heart J Acute Cardiovasc Care* 2014;3:354–62.
19. Machon M, Aldasoro E, Martinez-Camblor P, *et al.* Socioeconomic differences in incidence and relative survival after a first acute myocardial infarction in the Basque Country, Spain. *Gac Sanit* 2012;26:16–23.
20. Velders MA, James SK, Libungan B, *et al.* Prognosis of elderly patients with ST-elevation myocardial infarction treated with primary percutaneous coronary intervention in 2001 to 2011: a report from the Swedish Coronary Angiography and Angioplasty Registry (SCAAR) registry. *Am Heart J* 2014;167:666–73.
21. Alabas OA, Allan V, McLenachan JM, Feltbower R, Gale CP. Age-dependent improvements in survival after hospitalisation with acute myocardial infarction: an analysis of the Myocardial Ischemia National Audit Project (MINAP). *Age Ageing* 2014;43:779–85.
22. Tribouilloy C, Buiciuc O, Rusinaru D, Malaquin D, Lévy F, Peltier M. Long-term outcome after a first episode of heart failure. A prospective 7-year study. *Int J Cardiol* 2010;140:309–14.
23. Igland J, Vollset SE, Nygard OK, *et al.* Educational inequalities in 28 day and 1-year mortality after hospitalisation for incident acute myocardial infarction–a nationwide cohort study. *Int J Cardiol* 2014;177:874–80.
24. Kravdal O. A cancer survival model that takes sociodemographic variations in "normal" mortality into account: comparison with other models. *J Epidemiol Community Health* 2002;56:309–18.
25. Dickman PW, Coviello E. Estimating and modeling relative survival. *Stata J* 2015;15:186–215.

For peer review only - http://bmjopen.bmj.com/site/about/guidelines.xhtml

26. Human Mortality Database. University of California, Berkeley (USA), and Max Planck Institute for Demographic Research (Germany). Available at www.mortality.org (data downloaded on November 10, 2015).
27. Norwegian Institute of Public Health. Table: [D1: Deaths by sex, age, and detailed cause of death] D1: Dødsfall etter kjønn, alder og detaljert dødsårsak Secondary Table: [D1: Deaths by sex, age, and detailed cause of death] D1: Dødsfall etter kjønn, alder og detaljert dødsårsak 2015. Available at statistikkbank.fhi.no/dar/ (data downloaded on December 1, 2015). Norwegian
28. Strand BH, Steíngrimsdóttir ÓA, Grøholt EK, Ariansen I, Graff-Iversen S, Næss Ø. Trends in educational inequalities in cause specific mortality in Norway from 1960 to 2010: a turning point for educational inequalities in cause specific mortality of Norwegian men after the millennium? *BMC Public Health* 2014;14:1208.
29. Perme MP, Stare J, Esteve J. On estimation in relative survival. *Biometrics* 2012;68:113–20.
30. Coleman MP, Quaresma M, Berrino F, *et al.* Cancer survival in five continents: a worldwide population-based study (CONCORD). *Lancet Oncol* 2008;9:730–56.
31. Galobardes B, Shaw M, Lawlor DA, Lynch JW, Smith GD. Indicators of socioeconomic position (part 1). *J Epidemiol Community Health* 2006;60:7–12.
32. Mayer O, Jr., Simon J, Heidrich J, Cokkinos DV, De Bacquer D. Educational level and risk profile of cardiac patients in the EUROASPIRE II substudy. *J Epidemiol Community Health* 2004;58:47–52.
33. Cutler DM, Lleras-Muney A. Understanding differences in health behaviors by education. *J Health Econ* 2010;29:1–28.
34. Wagstaff A. The demand for health: theory and applications. *J Epidemiol Community Health* 1986;40:1–11.
35. Denvir MA, Lee AJ, Rysdale J, *et al.* Influence of socioeconomic status on clinical outcomes and quality of life after percutaneous coronary intervention. *J Epidemiol Community Health* 2006;60:1085–88.
36. Alter DA, Iron K, Austin PC, Naylor CD. Socioeconomic status, service patterns, and perceptions of care among survivors of acute myocardial infarction in Canada. *JAMA* 2004;291:1100–7.
37. Chan RH, Gordon NF, Chong A, Alter DA. Influence of socioeconomic status on lifestyle behavior modifications among survivors of acute myocardial infarction. *Am J Cardiol* 2008;102:1583–8.
38. Eloranta S, Lambert PC, Cavalli-Bjorkman N, Andersson TML, Glimelius B, Dickman PW. Does socioeconomic status influence the prospect of cure from colon cancer–a population-based study in Sweden 1965–2000. *Eur J Cancer* 2010;46:2965–72.
39. Huisman M, Kunst AE, Bopp M, *et al.* Educational inequalities in cause-specific mortality in middle-aged and older men and women in eight western European populations. *Lancet* 2005;365:493–500.
40. Wisloff T, Selmer RM, Halvorsen S, Fretheim A, Norheim OF, Kristiansen IS. Choice of generic antihypertensive drugs for the primary prevention of cardiovascular disease–a cost-effectiveness analysis. *BMC Cardiovasc Disord* 2012;12:26.

Figure legend

Figure 1. Patient survival and expected survival, according to years since diagnosis and educational group. The upper, nearly linear curves show expected survival rates, as calculated using the Ederer I method. The lower curves show patient survival rates, as estimated using the Kaplan-Meier method. Results are standardized for age and sex.

For peer review only

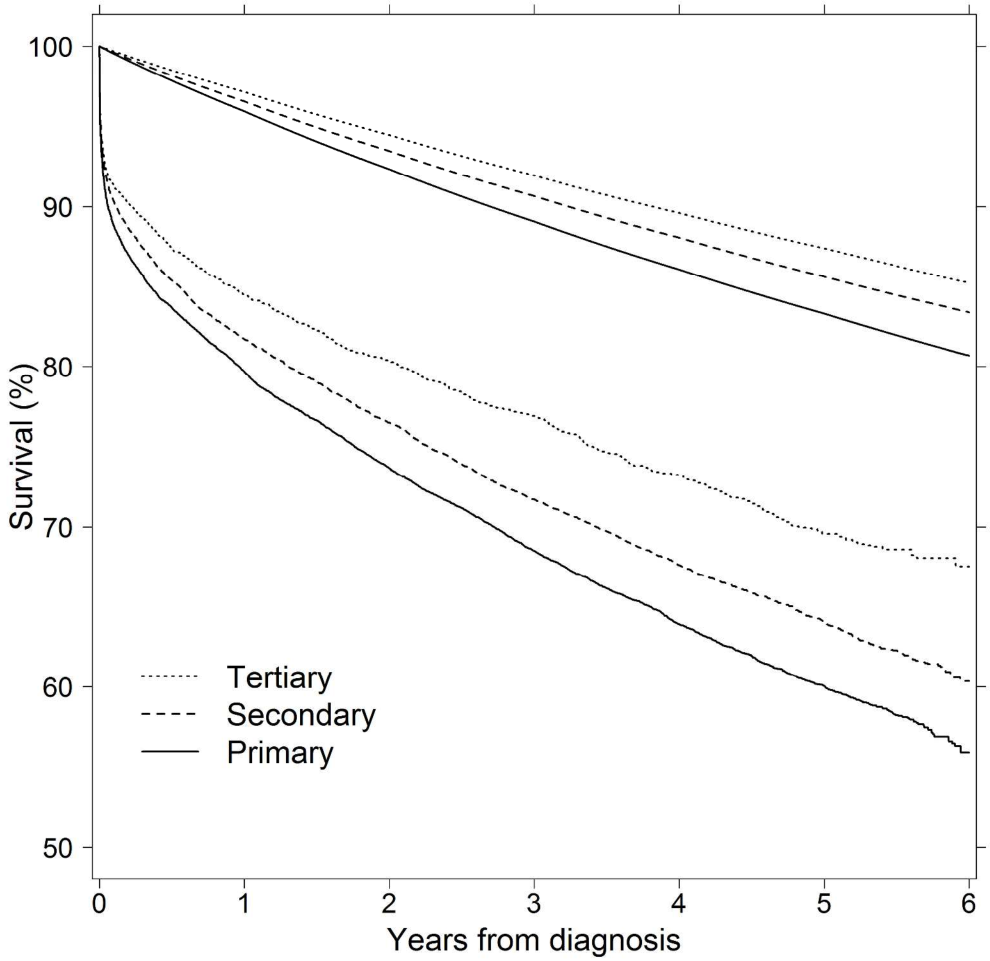

Figure 1. Patient survival and expected survival, according to years since diagnosis and educational group. The upper, nearly linear curves show expected survival rates, as calculated using the Ederer I method. The lower curves show patient survival rates, as estimated using the Kaplan-Meier method. Results are standardized for age and sex.

270x270mm (300 x 300 DPI)

ONLINE SUPPLEMENTARY MATERIAL

S1. Alternative calculation of education-specific expected survival

In our main analysis, we used Poisson regression to predict expected survival for the years 2011–2013. For comparison, we also applied an alternative method, based on an approach of Dickman et al. [1]. In this alternative method, all-cause mortality data obtained from the Human Mortality Database were adjusted based on our data containing educational levels.[2] Let $\mu_{g,t}$ denote the rate of all-cause mortality in year *t* and educational group *g*, and for brevity omit subscripts for age and sex. We then employed the following expression,

$$\mu_{g,t} = \mu_{c,t} \left(\frac{\mu_{g,t^*}}{\mu_{c,t^*}} \right)$$

where values for the whole country have group-subscripts replaced by *c*, and where t^* indicates that estimates for the years 2008–2010 were used. The values $\mu_{c,t}$ were obtained from the Human Mortality Database for each year *t = 2008, …, 2013*, while the standardized mortality ratio $\left(\mu_{g,t^*} / \mu_{c,t^*}\right)$ was taken from the obtained life tables on the general population from Statistics Norway for the combined period 2008–2010. The resulting estimates were nearly identical to those in our main analysis.

For peer review only

S2. Correction for infarction-related mortality and sensitivity analysis

Expected survival was corrected for mortality related to acute myocardial infarction, using the formula

$$p^* = p^{(1-\alpha)}$$

where p^* is the corrected survival probability, p is the observed survival probability, and α is the proportion of deaths due to acute myocardial infarction.[3] Because the necessary data were unavailable, we could not correct for α_g directly. Nonetheless, by emulating the relationship for overall cardiovascular mortality (ICD-10 I00–I99), as represented by λ, we obtained an approximate estimator:

$$\frac{\alpha_{g,t}}{\alpha_{c,t}} \approx \left(\frac{\lambda_{g,t}}{\mu_{g,t}}\right) \Big/ \left(\frac{\lambda_{c,t}}{\mu_{c,t}}\right) \quad \Leftrightarrow \quad \alpha_{g,t} \approx \alpha_{c,t} \left(\frac{\lambda_{g,t^*}}{\lambda_{c,t^*}}\right) \Big/ \left(\frac{\mu_{g,t^*}}{\mu_{c,t^*}}\right)$$

In the rightmost expression we used nationwide mortality data for $\alpha_{c,t}$.[4] The subscript t^* indicates that estimates for 2000–2010 were used for λ and μ.[5] The complex fraction was assumed to equal 1 in our main specification, and varying it based on previous findings had only a minor influence on the results.[5]

REFERENCES FOR THE SUPPLEMENTARY MATERIAL

1. Dickman PW, Auvinen A, Voutilainen ET, Hakulinen T. Measuring social class differences in cancer patient survival: is it necessary to control for social class differences in general population mortality? A Finnish population-based study. *J Epidemiol Community Health*. 1998;52:727–34.
2. Human Mortality Database. University of California, Berkeley (USA), and Max Planck Institute for Demographic Research (Germany). Available at www.mortality.org (data downloaded on November 10, 2015).
3. Talback M, Dickman PW. Estimating expected survival probabilities for relative survival analysis--exploring the impact of including cancer patient mortality in the calculations. *Eur J Cancer* 2011;47:2626–32.
4. Norwegian Institute of Public Health. Table: [D1: Deaths by sex, age, and detailed cause of death] D1: Dødsfall etter kjønn, alder og detaljert dødsårsak. Available at statistikkbank.fhi.no/dar/ (data downloaded on December 1, 2015). Norwegian.
5. Strand BH, Steíngrimsdóttir ÓA, Grøholt EK, Ariansen I, Graff-Iversen S, Næss Ø. Trends in educational inequalities in cause specific mortality in Norway from 1960 to 2010: a turning point for educational inequalities in cause specific mortality of Norwegian men after the millennium? *BMC Public Health* 2014;14:1208.

For peer review only - http://bmjopen.bmj.com/site/about/guidelines.xhtml

STROBE Statement—Checklist of items that should be included in reports of ***cohort studies***

	Item No	Recommendation	Reported on page #
**Title and abstract**	1	(*a*) Indicate the study's design with a commonly used term in the title or the abstract	Abstract p.2
		(*b*) Provide in the abstract an informative and balanced summary of what was done and what was found	Abstract p.2
**Introduction**			
Background/rationale	2	Explain the scientific background and rationale for the investigation being reported	Introduction p.4
Objectives	3	State specific objectives, including any prespecified hypotheses	Introduction, p.4
**Methods**			
Study design	4	Present key elements of study design early in the paper	In Abstract, p.2, and Methods, Data, p.5
Setting	5	Describe the setting, locations, and relevant dates, including periods of recruitment, exposure, follow-up, and data collection	Methods, Data, p.5
Participants	6	(*a*) Give the eligibility criteria, and the sources and methods of selection of participants. Describe methods of follow-up	Methods, Data, p.5
		(*b*) For matched studies, give matching criteria and number of exposed and unexposed	Not applicable
Variables	7	Clearly define all outcomes, exposures, predictors, potential confounders, and effect modifiers. Give diagnostic criteria, if applicable	Methods, Data, p.5
Data sources/ measurement	8*	For each variable of interest, give sources of data and details of methods of assessment (measurement). Describe comparability of assessment methods if there is more than one group	Methods, Data, p.5
Bias	9	Describe any efforts to address potential sources of bias	Methods, Statistical analyses, p.5/6
Study size	10	Explain how the study size was arrived at	Methods, Data, p.5
Quantitative variables	11	Explain how quantitative variables were handled in the analyses. If applicable, describe which groupings were chosen and why	Methods, Data, p.5 for education level; Methods, Statistical analyses, p.6/7 for survival outcomes
Statistical methods	12	(*a*) Describe all statistical methods, including those used to control for confounding	Methods, Statistical analyses, p. 6/7
		(*b*) Describe any methods used to examine subgroups and interactions	Not applicable
		(*c*) Explain how missing data were addressed	Methods, Data, p.5
		(*d*) If applicable, explain how loss to follow-up was addressed	Methods, Data, p.5
		(*e*) Describe any sensitivity analyses	Methods, Statistical analyses, p.6
**Results**			
Participants	13*	(a) Report numbers of individuals at each stage of	Methods, Data, p.5

For peer review only

For peer review only - http://bmjopen.bmj.com/site/about/guidelines.xhtml

		study—eg numbers potentially eligible, examined for eligibility, confirmed eligible, included in the study, completing follow-up, and analysed	
		(b) Give reasons for non-participation at each stage	Methods, Data, p.5
		(c) Consider use of a flow diagram	Not applicable
Descriptive data	14*	(a) Give characteristics of study participants (eg demographic, clinical, social) and information on exposures and potential confounders	Methods, Data, p.5, and Results, Table 1, p.10
		(b) Indicate number of participants with missing data for each variable of interest	Methods, Data, p.5
		(c) Summarise follow-up time (eg, average and total amount)	Methods, Data, p.5
Outcome data	15*	Report numbers of outcome events or summary measures over time	Results, Table 2, p.11
Main results	16	(*a*) Give unadjusted estimates and, if applicable, confounder-adjusted estimates and their precision (eg, 95% confidence interval). Make clear which confounders were adjusted for and why they were included	Results, Table 1, p. 10
		(*b*) Report category boundaries when continuous variables were categorized	Methods, Data, p.5, for attained education level
		(*c*) If relevant, consider translating estimates of relative risk into absolute risk for a meaningful time period	Not applicable
Other analyses	17	Report other analyses done—eg analyses of subgroups and interactions, and sensitivity analyses	Results, p.12
**Discussion**			
Key results	18	Summarise key results with reference to study objectives	Discussion, p.13
Limitations	19	Discuss limitations of the study, taking into account sources of potential bias or imprecision. Discuss both direction and magnitude of any potential bias	p. 15/16
Interpretation	20	Give a cautious overall interpretation of results considering objectives, limitations, multiplicity of analyses, results from similar studies, and other relevant evidence	p.12/13
Generalisability	21	Discuss the generalisability (external validity) of the study results	p.13/14
**Other information**			
Funding	22	Give the source of funding and the role of the funders for the present study and, if applicable, for the original study on which the present article is based	p.17

*Give information separately for exposed and unexposed groups.

Note: An Explanation and Elaboration article discusses each checklist item and gives methodological background and published examples of transparent reporting. The STROBE checklist is best used in conjunction with this article (freely available on the Web sites of PLoS Medicine at http://www.plosmedicine.org/, Annals of Internal Medicine at

For peer review only

For peer review only - http://bmjopen.bmj.com/site/about/guidelines.xhtml

http://www.annals.org/, and Epidemiology at http://www.epidem.com/). Information on the STROBE Initiative is available at http://www.strobe-statement.org.

For peer review only

For peer review only - http://bmjopen.bmj.com/site/about/guidelines.xhtml
